# Invited commentary: policies that support working parents and gender health equity—needed research and methodological challenges

**DOI:** 10.1093/aje/kwae223

**Published:** 2024-07-16

**Authors:** Alina Schnake-Mahl, Jaquelyn Jahn

**Affiliations:** Department of Health Management and Policy, Dornsife School of Public Health, Drexel University, Philadelphia, PA 19104, United States; Urban Health Collaborative, Dornsife School of Public Health, Drexel University, Philadelphia, PA 19104, United States; Department of Epidemiology and Biostatistics, Dornsife School of Public Health, Drexel University, Philadelphia, PA 19104, United States; Ubuntu Center on Racism, Global Movements, and Population Health Equity, Dornsife School of Public Health, Drexel University, Philadelphia, PA 19104, United States

**Keywords:** causal inference, paid leave, parental leave, employment policy, structural inequities

## Abstract

In a recent issue of the *Journal*, Platt et al (*Am J Epidemiol*. 2024;193(10):1362-1371) shed new light on the potential for supportive employment benefits, including family leave, flexible work hours, and employer-provided or subsidized childcare, to mitigate the risk of depression among full-time working mothers. The authors used a longitudinal study design and rigorous methods to carefully consider potential sources of bias, and, more broadly, their article underscores the importance of employment benefits as a social determinant of mental health for working mothers. In this commentary, we discuss some of the policy context surrounding employer benefits that support parenting, particularly around paid versus unpaid family leave laws and ordinances. We consider the ways in which the policy context affects larger structural inequities and the potential implications for internal and external validity.

**This article is part of a Special Collection on Mental Health**.

This article is linked to “Bringing home the benefits: do pro-family employee benefits mitigate the risk of depression from competing workplace and domestic labor roles?” (https://doi.org/10.1093/aje/kwae055).

##  

***Editor’s note:** The opinions expressed in this article are those of the authors and do not necessarily reflect the views of the* American Journal of Epidemiology.

## Introduction

Workplace policies supporting parenting are core to gender equity and substantially impact women’s mental and physical health, and population health broadly. These policies include paid and unpaid family leave, sick leave, and other policy areas in which the United States severely lags behind other nations. Within the United States, limited federal, state, and local laws create a tiered work–family support policy system, entrenching inequities in access and protection.^1^^,^^2^ Epidemiologic researchers in this area face the daunting task of handling bias related to whether, when, and under what conditions women work while parenting—issues that are not only are methodologically thorny but also central to policy and health equity. In a novel study published in a recent issue of the *Journal*, Platt et al^3^ tackled issues of bias head-on, in an analysis that examined the extent to which family-supportive employment benefits mitigate risk of depression among mothers who work full-time.

The authors addressed several biases that have challenged prior literature on employment, parenting, and health—reverse causation, selection, and time-varying confounding^4^—using marginal structural models applied to longitudinal data and careful sensitivity analyses. They found a higher depression risk among working mothers than among full-time workers without children, but crucially, these differential risks were reduced with access to family-supportive workplace benefits (family leave, flexible work scheduling, and childcare, termed “pro-family benefits” in the article) and after controlling for general employment benefits (eg, dental and health insurance, retirement benefits). The article addresses major biases that should be noted for future related research: (1) selection out of the full-time workforce based on having children and depression risk; (2) selection of parents (with lower depression risk) into jobs with family-supportive benefits; and (3) confounding by the job environment (eg, prestige, stability), even among full-time employed women. (A note on terminology: We use the term “women,” consistent with the original article, to reflect the sociocultural production of gender under patriarchy, and our use of this term is not meant to reinforce a binary or biologically essentializing understanding of gender.)

In addition to the paper’s methodological contributions, the work also illuminates the harmful effects of inequitable gender relations that are reinforced, or mitigated, by employment policy. Depression among women is a serious and persistent public health problem,^5^ and rigorous research identifying social drivers of women’s depression can help encourage preventive policies that strengthen gender equity.^6^ Understanding social and structural determinants of women’s depression adds context and complexity to biomedical understandings of sex differences in mental health outcomes and perinatal depression.^7^^,^^8^ The article’s focus on “competing roles” (ie, women parenting while working full-time) also highlights inequities in domestic work, particularly among heterosexual parents—a continued challenge for gender equity and women’s mental health.^9^

In this commentary, we situate Platt et al’s analysis within the broader context of workplace and social safety-net policies. We consider how the policy context and larger structural inequities, including structural racism and economic inequity, affect parental employment and access to workplace benefits. We give 3 examples of ways in which policies and structural inequity (and their interactions) could be incorporated into future work and discuss potential challenges for internal and external validity.^10^

## Incorporating policy and equity

### Example 1: policy context—paid and unpaid family leave

The United States stands alone among high-income countries in its lack of federal *paid* family leave.^11^ It does have a required *unpaid* family and medical leave law, through the 1993 Family Medical Leave Act (FMLA), but paid versus unpaid family and medical leave are distinct categories of employment benefits regulated by separate laws, ordinances, and benefit structures.^12^ The Platt et al article, however, does not distinguish between these two types of benefits. *Unpaid* family leave prevents employees from being fired for birth, adoption, or serious illness or injury, while *paid* family leave covers the same events, but with pay.^12^ Access to these benefits differs dramatically: In 2023, 90% of civilian workers (workers in private industry or state or local government) had access to unpaid family leave, while only 27% had access to paid family leave.^13^

The FMLA improved access to unpaid family leave, but as of 2023 10% of civilian workers were excluded from FMLA coverage because they worked at small businesses, were part-time employees (<24 hours/week), or had less than 1 year of tenure.^14^ Since 2000, access to *paid* family leave has grown as states and cities have enacted paid family leave laws, but a patchwork of coverage remains.^2^^,^^15^ As of early 2024, 14 states and a few cities have enacted mandatory paid family leave for private sector employees.^16^ Some private employers in places without mandates provide these benefits voluntarily, but this is less common in industries with large numbers of low-wage workers and among nonunionized workers.^13^^,^^17^

Platt et al constructed a family-supportive benefits index using self-reported access to several benefits (family leave, flexible scheduling, childcare) in a national sample of full-time working women. This approach allows for inferences about this cluster of supportive policies, which may have mutually beneficial effects (eg, the mental health benefits of family leave may be heightened among those with flexible scheduling) that are less easily examined in econometric studies of the maternal health effects of single benefits mandated by law.^4^^,^^18^^,^^19^ Policy analyses that focus on jurisdiction-level policy implementation also often lack the precision of individual-level benefit access.

However, Platt et al’s family-supportive benefits measure does not distinguish between *paid* and *unpaid* family leave, and doing so could arguably make the analysis more meaningful for policy translation. To our knowledge, data on paid and unpaid leave are available in the National Longitudinal Survey of Youth.^20^ The policy analysis literature suggests that paid and unpaid family leave have differential magnitudes and durations of impact on mental health,^21^ and understanding whether these differential benefit effects operate at the individual level is important. These differences may be particularly acute for low-income workers, who may be unable to use family leave unless it is paid.^22^^,^^23^ Including both paid and unpaid leave in the family-supportive benefits measure likely underestimates the potential mitigating effects of paid leave on maternal depression risk.

Future research should bring in paid versus unpaid policy distinctions to inform a more well-defined measure of benefits^24^ that align with existing paid family leave policies. This alignment can improve the research’s generalizability and effectiveness in contributing to evidence on the health impacts of paid and unpaid family leave policies, shifting the work from consideration of a social determinant of mental health (access to employment benefits) to consideration of a structural determinant, policies, and laws.^10^^,^^25^

### Example 2: expanding to additional policies that support working parents

Working parents need a variety of policies and benefits to support them, but our current policy context affords them very little. Platt et al’s analysis includes 3 types of family-supportive benefits: family leave, flexible work benefits, and employer-provided or sponsored childcare. The latter two are uncommon: In 2023, only 12% of the civilian worker population had access to workplace-provided/sponsored childcare and only 9% to flexible work programs.^13^

We encourage future researchers to investigate a wider variety of supportive policies. For example, sick leave, or sick time, provides for paid short-term coverage for illness, injury, or care for individuals and their families,^12^ making paid sick leave a core family-supportive benefit for employees’ and their families’ mental health.^21^ Paid sick leave data are also available in the National Longitudinal Survey.^20^ Similar to paid family leave, there is no federal paid sick leave law (though there was briefly one during the first year of the COVID-19 pandemic^26^). As of early 2024, 15 states, the District of Columbia, and a number of cities and counties had paid sick leave mandates.^27^ Together with voluntary employer coverage, these laws and ordinances have increased paid sick leave coverage, but millions of American workers remain without access.^13^ Future research should integrate paid sick leave as an additional family-supportive benefit and examine its cumulative and interactive effects with other benefits.

Additional policies, such as minimum wage, postpartum and expanded Medicaid eligibility, and abortion access laws, are also arguably family-supportive and prohealth policies^28^ because they have enormous significance for the financial and physical health of working parents and their families. These policies do not regulate employment benefits, but consideration of the role of such policies in mitigating maternal depression is particularly important in the post–*Roe v. Wade* era, when state legislatures and state supreme courts are increasingly deciding who becomes a parent and who can access benefits like paid time off.^15^^,^^29^ For example, none of the states that ban or severely restrict abortion access have paid family leave laws.^15^ A separate category of policies around unionization and collective bargaining also substantially affect employees’ receipt of family-supportive benefits and are of particular importance in states that lack many of these more health-oriented family-supportive policies. Consideration of this inequitable geographic distribution of family-supportive policies has important implications for causal inference, as this differential exposure distribution may generate positivity violations (eg, all or no workers with access to specific benefits) or act as a potential confounder. In many of the states that lack paid family leave, Medicaid eligibility has not been expanded, workers cannot unionize, and the minimum wage remains $7.25 an hour,^28^ producing additional inference challenges related to policy co-occurrence and clustering.^19^ Understanding the policy context regulating family-supportive benefits and examining the distribution of laws across states, cities, and subpopulations can help researchers avoid, or at least be aware of, these potential challenges in both individual-level analyses and policy evaluations.

### Example 3: inequity in employment and employment benefits

In the United States, occupational segregation contributes to differential access to employment and workplace protections and subsequent health inequities.^30^^,^^31^ Because of ongoing and historical structural racism in the United States, including that operating through inequitable employment opportunities and racist employment policies and practices, racially minoritized women are overrepresented among low-wage workers, while White men are overrepresented in high-wage employment.^31^ Low-wage workers, workers of color, disabled workers, and single parents are also less likely to work full-time and to have access to various employment benefits, including more limited access to paid leave (sick and family) and workplace flexibility.^32-35^ For example, as of 2023, only 6% of the lowest-wage workers had access to paid family leave versus 48% of the highest-wage workers.^36^ There are also substantial inequities in LGBTQI+ access to important workplace benefits; only 37% of LGBTQI+ workers had access to paid sick or family and medical leave in 2023.^37^

The findings from Platt et al’s study likely have important implications for structural inequities, though these hypotheses could not be tested directly. The authors restricted the analysis to full-time employed women to avoid structural positivity violations related to limited benefits access among part-time workers. They also addressed potential confounding due to occupational segregation through adjustment for differential employment structures—for example, by adjusting for nonfamily benefits and race/ethnicity (a social construct), alongside wages and hours worked. While their approach strengthens internal validity, it excludes populations least likely to have but most likely to benefit from family-supportive workplace policies.^38-40^ No single study can or should examine all sources of effect modification and inequity, but future work should aim to better understand, and ultimately prevent, adverse health consequences of occupational segregation and differential access to employment benefits.

Moreover, as Platt et al pointed out, future researchers will have to grapple with selection bias from parents leaving the workforce or switching to part-time work (or multiple part-time jobs) as a consequence of parenthood without supportive social policies. Women are more likely to entirely leave the work force absent family-supportive benefits including flexible work hours^41^ and paid and unpaid family leave laws,^22^^,^^39^ though the effects are weaker if the leave is unpaid.^38^^,^^42^ To bolster the scientific evidence base in this area and inform equitable policy-making,^6^ future work can apply causal inference approaches^43^ with intersectional understandings of the ways in which structural racism and sexism, along with racial capitalism, jointly produce inequities in women’s mental health.^44^^,^^45^

## Conclusion

We commend the authors on their article, as it will surely be foundational to future work on gender equity and workplace health. Their approach to identifying and addressing bias related to parenting, employment, and mental health attends to long-standing gaps in this research area and is necessary for future rigorous work. We hope that in subsequent work, investigators can delve into the importance of policy contexts and occupational segregation in women’s health and health equity. Our recommendations apply to research involving benefit coverage but are also relevant for other individual-level causal research that involves exposures or mediators whose distributions are largely policy-dependent. Together such work can advance our understanding of the causal effects of individual exposures and effect modifiers, and ensure that findings connect directly to policies that support working people and families.

## Data Availability

No data were used for this article.
